# Regulation of an endophytic nitrogen-fixing bacteria GXS16 promoting drought tolerance in sugarcane

**DOI:** 10.1186/s12870-023-04600-5

**Published:** 2023-11-17

**Authors:** Qian Nong, Li Lin, Jinlan Xie, Zhanghong Mo, Mukesh Kumar Malviya, Manoj Kumar Solanki, Zeping Wang, Xiupeng Song, Yangrui Li, Changning Li

**Affiliations:** 1 Key Laboratory of Sugarcane Biotechnology and Genetic Improvement (Guangxi), Ministry of Agriculture and Rural Affairs, Guangxi Key Laboratory of Sugarcane Genetic Improvement, Nanning, 530007 Guangxi China; 2 Guangxi Key Laboratory of Biology for Crop Diseases and Insect Pest, Plant Protection Research Institute, Guangxi Academy of Agricultural Sciences, Nanning, 530007 Guangxi China; 3 Institute of Biological Science, Sage University Indore, Bhopal, Madhya Pradesh India; 4 Plant Cytogenetics and Molecular Biology Group, Institute of Biology, Biotechnology and Environmental Protection, Faculty of Natural Sciences, University of Silesia in Katowice, 40-032 Katowice, Poland

**Keywords:** Sugarcane, Endophytic nitrogen-fixing bacteria, Drought tolerance, Transcriptome, Gene regulatory network

## Abstract

**Background:**

Drought limits crop growth and is an important issue in commercial sugarcane (*Saccharum officinarum*) production. Drought tolerance in sugarcane induced by endophytic nitrogen-fixing bacteria is a complex biological process that ranges from altered gene expression and cellular metabolism to changes in growth and productivity.

**Results:**

In this study, changes in physiological features and transcriptome related to drought tolerance in sugarcane conferred by the *Burkholderia* endophytic nitrogen-fixing bacterial strain GXS16 were investigated. Sugarcane samples inoculated with GXS16 exhibited significantly higher leaf relative water content than those without GXS16 inoculation during the drought stages. Sugarcane treated with GXS16 had lower levels of H_2_O_2_ and higher levels of abscisic acid than sugarcane not treated with GXS16 in the non-watering groups. Transcriptomic analysis of sugarcane roots identified multiple differentially expressed genes between adjacent stages under different treatments. Moreover, both trend and weighted correlation network analyses revealed that carotenoid biosynthesis, terpenoid backbone biosynthesis, starch and sucrose metabolism, and plant hormone signal transduction strongly contributed to the drought-tolerant phenotype of sugarcane induced by GXS16 treatment. Accordingly, a gene regulatory network including four differentially regulated genes from carotenoid biosynthesis (*crtB*, *crtZ*, *ZEP* and *CYP707A*) and three genes from terpenoid backbone biosynthesis (*dxs*, *dxr*, and *PCME*) was constructed.

**Conclusions:**

This study provides insights into the molecular mechanisms underlying the application of GXS16 treatment to enhance drought tolerance in sugarcane, which will lay the foundation for crop development and improve productivity.

**Supplementary Information:**

The online version contains supplementary material available at 10.1186/s12870-023-04600-5.

## Background

Sugarcane is a unique crop that is an important source of raw materials for sugar and bioethanol production worldwide. The global harvest area was approximately 26 million hectares in 2016, with approximately 40% of the world’s production taking place in Brazil, which is followed by India (18.4%) [1]. In China, the major sugarcane planting and sugar-producing areas are located between the latitudes 18.5° to 32°N and longitudes 92° to 122°E (Guangxi, Yunnan, Guangdong, Hainan, Fujian, Taiwan, Zhejiang, Sichuan, Guizhou, Hunan, and Jiangxi Provinces). Guangxi contributes to more than 60% of the sugarcane and sugar production in China and is the third largest sugar-producing region in the world after Brazil and India [2]. However, plant growth and crop productivity of sugarcane are affected by unfavorable climatic conditions and environmental stresses, such as drought, salinity, submergence conditions, heavy metal toxicity, cold stress, and high temperatures, which increase in frequency and intensity. Drought is the most deleterious abiotic stressor that threatens plant growth, crop production, and agricultural development. For example, water scarcity is responsible for progressive losses (17–52%) in sugarcane yield (tonnes of cane per hectare) [3].

The direct negative effects of drought stress on plants include morphological, physiological, and chemical responses based on the genotype [4], gene expression [5, 6], plant growth period [7], drought duration (rapid or gradual) and intensity (severe or mild) [8]. Growth is the result of daughter cell production by meristematic cell division and the subsequent massive expansion of young cells. Decreased plant height, stem diameter, and leaf area caused by impaired mitosis are the main morphological responses of plants to increasing drought stress, which are caused by impaired mitosis [9]. Abscisic acid (ABA), cytokinins, ethylene, and malate have been implicated in the root–shoot signalling and contribute to stomatal closure [10]. Simultaneously, the gas exchange parameters of plants are hampered, which could be due to decreased leaf expansion, premature leaf senescence, decreased chlorophyll content, and changes in the structures of pigments and proteins [11]. Moreover, water deficit induces oxidative stress in plants by causing overproduction of reactive oxygen species (ROS), such as superoxide (O^2−^), hydrogen peroxide (H_2_O_2_), and •OH radicals, which can directly attack membrane lipids and increase lipid peroxidation [12].

Plant growth-promoting characteristics and environmental stress-resistant phenotypes include biological nitrogen fixation (BNF), phosphate solubilization, mineral uptake, and siderophore and phytohormone production. For example, nitrogen-fixing microorganisms generally encode nitrogenases that are highly conserved and regulated by *nifH*, *nifD*, *nifK* and *nifS* genes [13, 14]. The BNF process, which involves several physiological and biochemical modifications in crop tissues, can convert N_2_ into a plant-usable form (inorganic nitrogen-containing compounds), such as ammonia (NH_3_), and contribute to the growth, production, development, and drought tolerance of sorghum [15], rice [16], pea [17], cucumber [18, 19], pepper [20], wheat [21], maize [22], and other plants in arable land and natural ecosystems. In the sugarcane cv.SP70-1143, gene expression during the water deficit assay appeared to be changed by colonization with the diazotroph Gluconacetobacter diazotrophicus PAL5 to correspond to particular ABA-dependent reactions and overcome drought stress [23]. Thus, nitrogen-fixing microorganisms have attracted attention for improving sugarcane drought tolerance, which could promote crop production, decrease the use of synthetic nitrogen fertilizers, and achieve global agro-environmental sustainability. However, drought tolerance of sugarcane induced by endophytic nitrogen-fixing bacteria is a complex biological process, ranging from altered gene expression and cellular metabolism to changes in drought tolerance phenotype, growth, and productivity.

In this study, we explored the application of GXS16 to promote the response of sugarcane plants to drought stress. We performed physiological measurements, metabolite profiling, and transcriptomic analysis to investigate the mechanisms underlying drought tolerance in sugarcane treated with GXS16 at three different stages. Dozens of differentially accumulated metabolites (DAMs) and differentially expressed genes (DEGs) in structural roots were identified, and relevant molecular mechanisms and pathways were comprehensively elucidated. The data from this study provide valuable information for further application and development of GXS16 to enhance drought tolerance in sugarcane and provide new insights for increasing crop yields.

## Results

### Physiological measurement of sugarcane

**Fig. 1:**
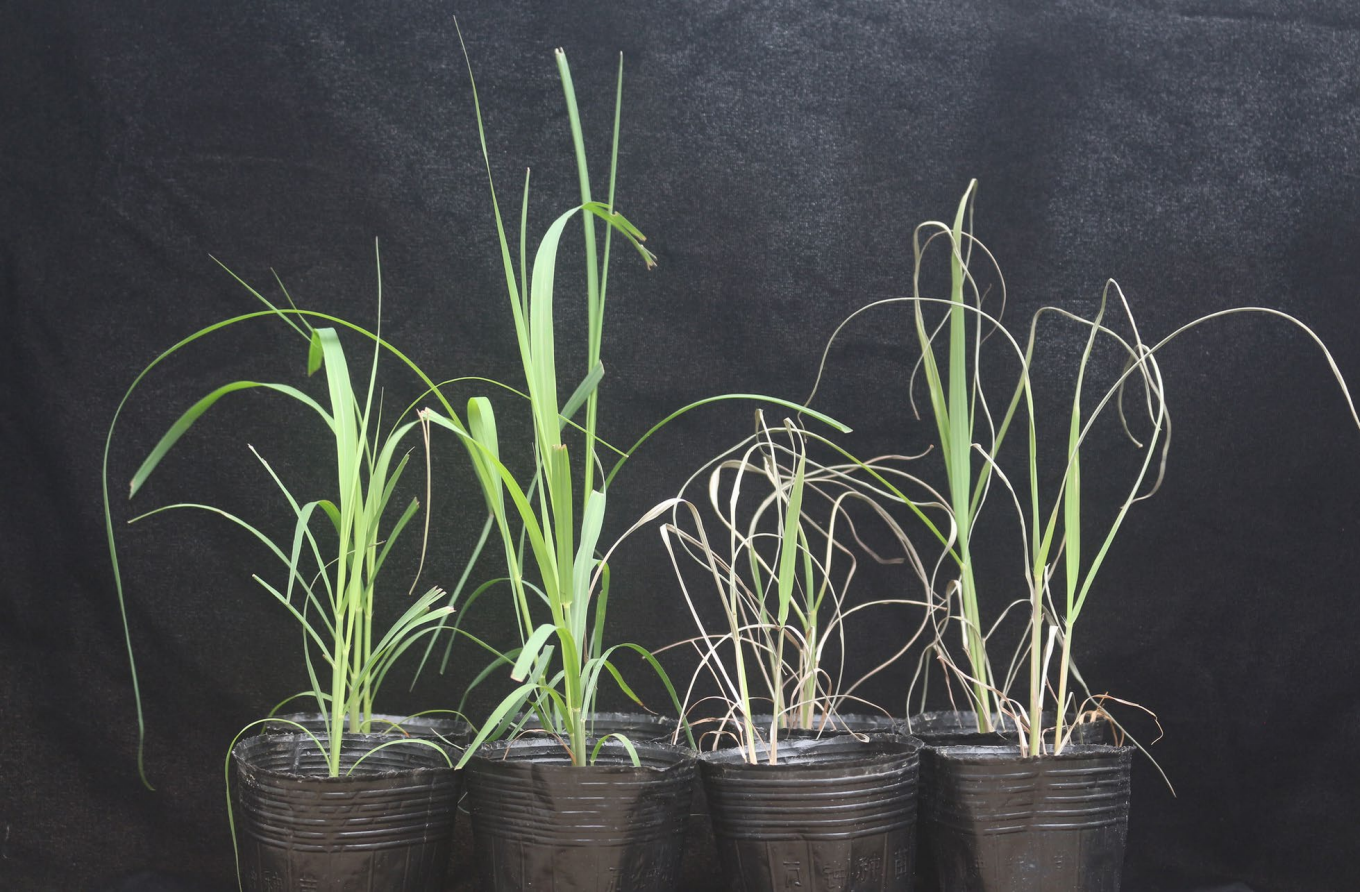
Photographs of sugarcane plants in different treatment groups, including control (C), inoculation control (IC), drought (D), and inoculation drought (ID) groups, from left to right

The 1-aminocyclopropane-1-carboxylate (ACC) deaminase activity of nitrogen-fixing bacteria GXS16 was determined to be 15.37 μmoL α-ketobutyrate/(mg protein·h). The copy number of GXS16 in the sugarcane roots increased significantly after GXS16 inoculation and increased gradually during the experimental stages (Fig. S1). Treatment with the nitrogen-fixing bacteria GXS16 largely alleviated the drought effects on sugarcane plants (Fig. 1), and the inoculation groups exhibited relatively higher plant height than the control group (Table S1).

**Fig. 2:**
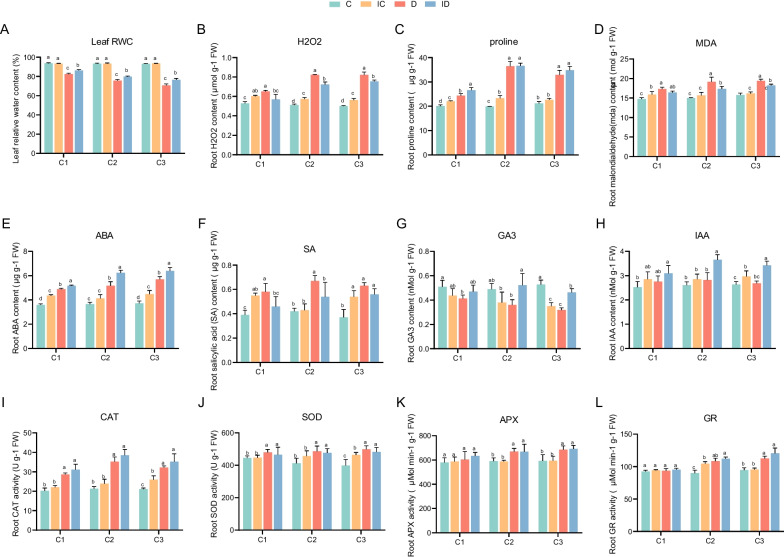
Measurements of physiological indices for different groups across drought stages: (**A**) Leaf RWC; (**B**) H_2_O_2_ level; (**C**) Proline level; (**D**) Malondialdehyde (MDA) level; (**E**) Abscisic acid (ABA) level; (**F**) Salicylic acid (SA) level; (**G**) Gibberellic acid (GA3) level; (**H**) Indole acetic acid (IAA) level; (**I**) Catalase (CAT) activity; (**J**) Superoxide dismutase (SOD) activity; (**K**) Ascorbate peroxidase (APX) activity; (**L**) Glutathione reductase (GR) activity. Comparisons were performed using ANOVA with Duncan’s multiple-range test. c1, c2, and c3 on the x-axis indicate the three stages at 3 days (D1), 5 days (D2), and 7 days (D3) after the water deficit, respectively

Physiological indices, including leaf relative water content (RWC, Fig. 2A), H_2_O_2_ (Fig. 2B), proline (Pro, Fig. 2C), malondialdehyde (MDA, Fig. 2D), ABA (Fig. 2E), salicylic acid (SA, Fig. 2F), the levels of ABA and gibberellin (GA3, Fig. 2G), indole-3-acetic acid (IAA, Fig. 2H), activities of catalase (CAT, Fig. 2I), superoxide dismutase (SOD, Fig. 2J), ascorbate peroxidase (APX, Fig. 2K), and glutathione reductase (GR, Fig. 2L), exhibited significant changes across the experimental groups (Fig. 2). Notably, leaf RWC in the non-watered group was significantly lower compared with the control group, whereas leaf RWC in the non-watered group treated with GXS16 was significantly higher compared with the non-watered group without GXS16 treatment (Fig. 2A), indicating a difference in drought resistance induced by the addition of probiotic GXS16. Although the H_2_O_2_ content increased in GXS16-treated sugarcane in the non-watered groups, the opposite results were observed in the watering groups (Fig. 2B). The average ABA levels in each group decreased in the following order: inoculation drought (ID) > drought (D) > inoculation control (IC) > control (C) at the same drought stage (Fig. 2E).

### Transcriptome analysis of sugarcane at different drought tolerance stages

**Fig. 3:**
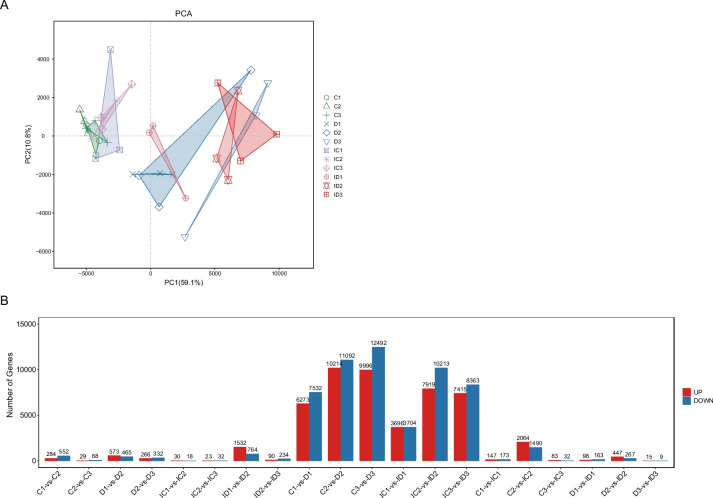
Global gene expression profiling. **A** Principal component analysis (PCA) of all RNA-seq samples. Different shapes of the points represent different experimental groups. **B** Number of differentially expressed genes (DEGs) in all comparisons. Red and blue bars indicate upregulated and downregulated genes, respectively

For transcriptome profiling, an average of 6.6 million reads per sample were sequenced (Sequence Read Archive accession no.PRJNA951683). An average of 6.5 million reads per sample, with a Q20 percentage above 95%, were obtained after quality filtering (Table S2). The GC content of the clean reads was approximately 55.18–57.11% (Table S2). Principal component analysis (PCA) revealed that the replicates were similar (Fig. 3A). Specifically, the samples from the non-watered and control groups were well separated (Fig. 3A).

**Fig. 4:**
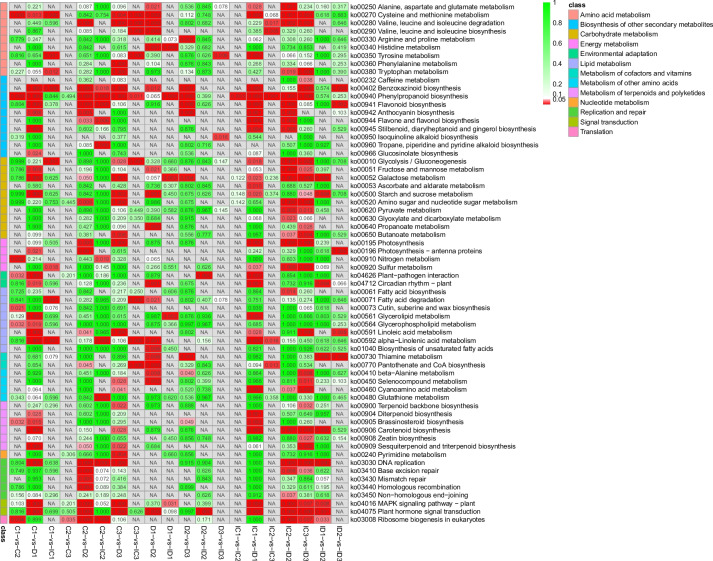
Heat map illustrating the q-value of KEGG pathway enrichment of DEGs for different comparisons. Red (q-value < 0.05) and green (q-value > 0.05) squares represent significantly and insignificantly enriched pathways, respectively. NA indicates that no DEGs were categorized into that pathway

Several DEGs identified in the comparisons of C1-Vs-IC1, C2-Vs-IC2, C3-Vs-IC3, D1-Vs-ID1, D2-Vs-ID2, and D3-Vs-ID3 revealed substantial changes in gene expression induced by GXS16 treatment under the same drought tolerance conditions (Fig. 3B and Table S3). The biosynthesis of other secondary metabolite pathways (especially phenylpropanoid and benzoxazinoid biosynthesis) were significantly enriched in the DEGs of the different comparative groups (Fig. 4). However, the pathways involved in drought tolerance induced by GXS16 treatment were not identified when the two groups were compared (Fig. 4). The global gene expression results also revealed that several genes were significantly affected by drought stress in the same treatment groups (with and without GXS16 treatment) (Fig. 3B and Table S3).

### Comparison trends of different drought tolerance levels of sugarcane

Based on the STEM software, trend analysis was performed on the transcriptome data across the three stages (3, 5, and 7 days) for C (Fig. S2A), D (Fig. S2B), IC (Fig. S2C) and ID groups (Fig. S2D). Three significant profiles were identified for each group and divided into four trends: upward or downward and trends with peaks or valleys at 5 d (Fig. S2).

**Fig. 5:**
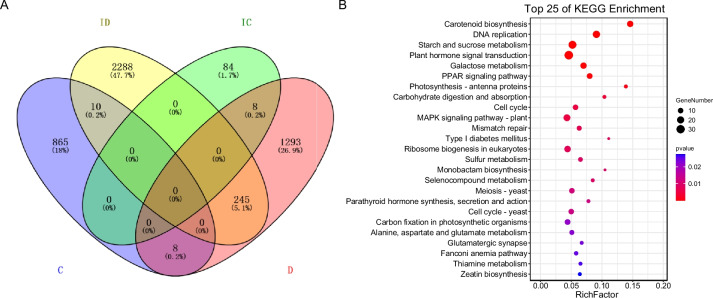
Statistics and functional analysis of trend gene sets. **A** Venn diagram illustrating common and unique gene sets with a particular trend in different groups. **B** KEGG pathway enrichment analysis of genes with a distinct trend in the ID group compared with the other three groups

The gene sets in the significant profiles were compared to identify the distinctive genes for each group. Among these, 2,288 and 1,293 unique genes in the D and ID groups, respectively, were identified as distinctive genes with a significant trend during drought stress with and without GXS16 treatment (Fig. 5A). The distinctive genes that exhibited a particular trend (any upward or downward trend, and trends with peaks or valleys) in the ID group but not in the other three groups were further identified as specific gene sets representing expression level differences during drought stress with GXS16 treatment, which were related to the promotion of drought tolerance (Fig. 5A). This gene set was then subjected to KEGG pathway enrichment analysis (Fig. 5B). The most significantly enriched pathways were carotenoid biosynthesis, starch and sucrose metabolism, and plant hormone signal transduction (Fig. 5B).

### Weighted gene co-expression network analysis of sugarcane at different stages of drought tolerance

**Fig. 6:**
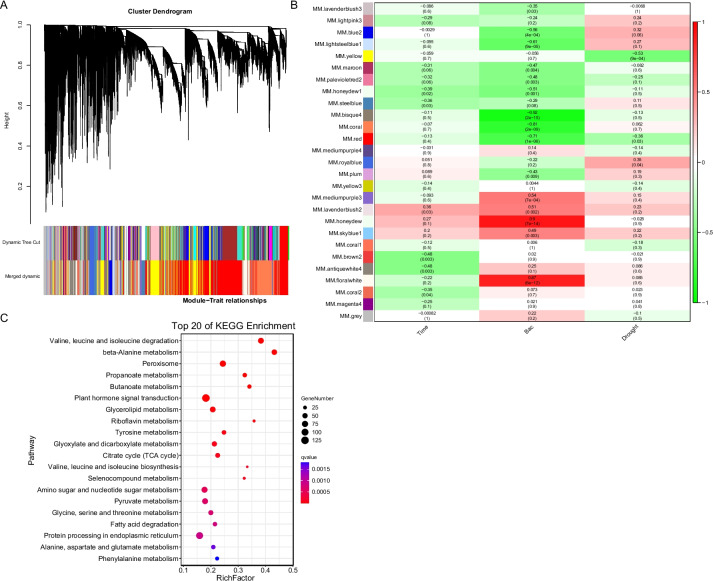
Gene co-expression analysis using Weighted correlation network analysis (WGCNA). **A** Gene dendrogram after the dynamic tree cut. The merged dynamic method was used to merge similar gene modules. Colors of the modules correspond to those of the merged gene dendrogram in (**A**). **B** Heat map showing module trait relationships. Numbers in each rectangle indicate the correlation coefficients between each module and trait, with the corresponding *P*-values in parentheses. Red and green represent positive and negative correlations, respectively. **C** KEGG analysis of genes in the honeydrawn module from WGCNA

Weighted correlation network analysis (WGCNA) identified 27 modules of co-expressed genes (Fig. 6A). Module-trait correlation analysis was conducted to identify the significant gene modules correlated with traits, and the honeydrawn module showed a significant positive correlation with the inoculation of the bacterium GXS16 (Fig. 6B). Specific trend gene sets in the ID group (Fig. 6A) matched those in the coexpression modules (Table S4). Among these, 888 genes were identified in honeydrawn modules (Table S4). KEGG pathway enrichment analysis of these key genes revealed that photosynthesis, starch, sucrose selenocompound, galactose, sulfur, pyruvate, alanine, aspartate, glutamate, and thiamine metabolism, MAPK signaling pathway, plant hormone signal transduction, and carotenoid and terpenoid backbone biosynthesis were significantly enriched and overlapped with those enriched for specific trend gene sets in the ID group (Fig. 6C and Table S5).

**Fig. 7:**
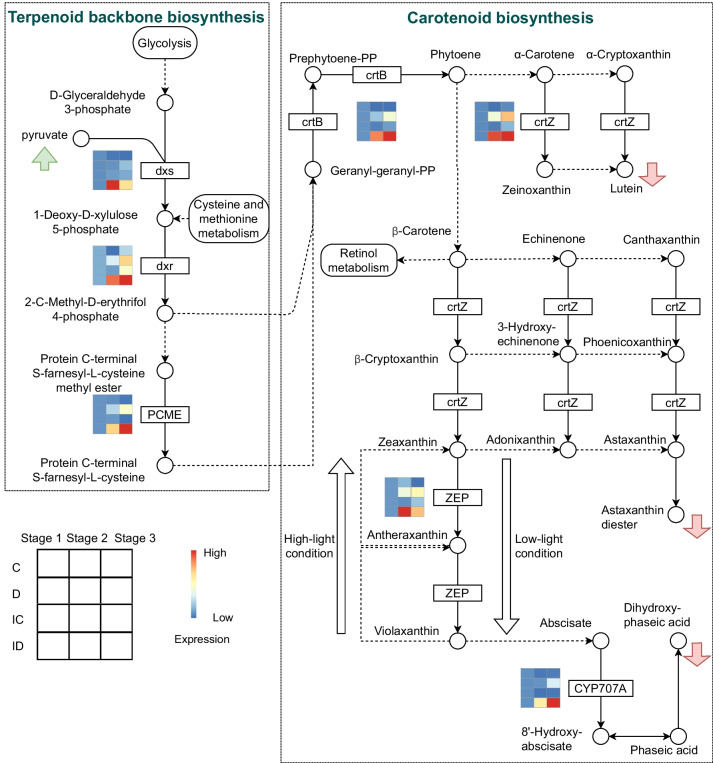
Key pathways involved in drought tolerance induced by GXS16 treatment [24]. The expression levels of genes in the honeydrawn module across stages for the C, D, IC, and ID groups are shown in the heat maps. Green and red arrows indicate the upstream and downstream pathways, respectively

ABA is an important signaling molecule derived from the carotenoid biosynthesis pathway, which utilizes geranyl–geranyl-PP from the terpenoid backbone (Fig. 7). Among the genes in honeydrew module, four genes namely *crtB* (15-cis-phytoene synthase), *crtZ* (beta-carotene 3-hydroxylase), *ZEP* (zeaxanthin epoxidase), and *CYP707A* (( +)-abscisic acid 8'-hydroxylase) participated in carotenoid biosynthesis and three genes namely *dxs* (1-deoxy-D-xylulose-5-phosphate synthase), *dxr* (1-deoxy-D-xylulose-5-phosphate reductoisomerase), and *PCME* (prenylcysteine alpha-carboxyl methylesterase) participated in terpenoid backbone biosynthesis. These were upregulated during drought stress in the ID group (Fig. 7). *crtB*, *crtZ, CYP707A, dxr*, and *PCME* were gradually upregulated across the stages in the ID group, whereas *ZEP* and *dxs* peaked at 5 days (Fig. 7). In comparison, changes in the expression of these genes were not evident in the other groups (Fig. 7). The increased ABA levels induced by the upregulation of these genes were closely related to the promotion of drought tolerance by GXS16 treatment.

## Discussion

Recently, the interactions between plants and endophytic nitrogen-fixing microorganisms have emerged as interesting insights that can be applied to novel and sustainable agricultural practices. Utilization of these microorganism,, which survives in crop tissues, is one of the most suitable technologies for improving crop growth and drought stress resistance. They release various phytochemicals, including ABA, IAA, ACC deaminase, and various volatile compounds that enhance drought tolerance [25]. In our research, the ACC deaminase activity of GXS16 was determined to be 15.37 μmoL α-ketobutyrate/(mg protein·h) (Fig. S1), which can consequently decrease the production of ethylene by hydrolyzing the biosynthetic precursor for ethylene (ACC) and producing ammonia and α-ketobutyrate [26–28]. This pathway reduces ethylene accumulation in sugarcane under adverse drought-induced conditions, thereby promoting sugarcane development.

The defense mechanism of sugarcane under drought stress acts by maintaining water content to the maximum possible extent to protect cellular structures. In this study, the leaf RWC level of the sugarcane variety ROC16 in group D was lower than 80.0% after five days of incubation under drought conditions and was significantly lower than that of group ID (Fig. 2A). Similarly, leaf RWC levels of sugarcane TCP87-3388 and HOCP93-776 (drought-susceptible crops) decreased to approximately 80–85% after 45 days and 73–79% after 90 days of drought stress conditions [29]. It is reported that RWC ranged from 45 to 58% for barley, 56% to 72% for wheat, and around 65% for corn [30, 31]. Therefore, to develop novel breeding techniques, it is necessary to better understand the processes by which GXS16 increases the drought tolerance of ROC16. Vargas et al. showed that sugarcane SP70-1143 died under drought stress at 1 month, while SP70-1143 inoculated with the diazotroph Gluconacetobacter diazotrophicus PAL5 still survived [23]. ABA is a stress-responsive hormone that is crucial for the detection of and response to drought. ABA concentration was also significantly higher in the ID group than in the D group. Our previous study showed that direct foliar application of ABA resulted in the accumulation of ABA in sugarcane leaves [32] and further improved their drought tolerance by continuously triggering the overexpression of the antioxidant defense system (Fig. 2E). Although H_2_O_2_ content in group ID was significantly higher than that in group C, our data indicated that GXS16 inoculation enhanced the drought tolerance of sugarcane by reducing H_2_O_2_ concentration (Fig. 2B). In C4 plants, H_2_O_2_ is generated in chloroplasts and is harmful to cellular organelles and membranes [33]. It also acts as a secondary messenger in signal transduction because of its relatively long half-life and high membrane permeability [33]. In addition, CAT was significantly upregulated compared with group D, which indicated that CAT and not SOD act to reduce H_2_O_2_ content in sugarcane under drought conditions (Fig. 2I) and provide protection against oxidative damage [12].

To further explore the critical DEGs and metabolic pathways involved in sugarcane development under drought stress, we performed trend analysis and WGCNA to analyze the expression modules of genes responding to drought stress. Both analyses revealed that carotenoid and terpenoid backbone biosynthesis, starch and sucrose metabolism, and plant hormone signal transduction contribute to the drought-tolerant phenotype of sugarcane promoted by GXS16 treatment. Terpenoids are the most diverse group of natural products, encompassing over 65,000 structures that are involved in protecting plants from abiotic environments [34]. Two biosynthetic pathways, mevalonate (MVA) and 2-C-methyl-D-erythritol 4-phosphate/1-deoxy-D-xylulose 5-phosphate (MEP/DOXP), are involved in the production of two basic building blocks: isopentenyl diphosphate (IPP) and dimethylallyl diphosphate (DMAPP) [35]. Prenyltransferases can generate higher-order building blocks, such as geranyl diphosphate (GPP), farsenyl diphosphate (FPP), and geranylgeranyl diphosphate (GGPP), which are monoterpenoid, sesquiterpenoid, and diterpenoid, respectively [35]. Combined with the results of our study, the upregulated *dxr* and downregulated *dxs* are regulatory factors in the MEP/DOXP pathway for drought tolerance in sugarcane (Fig. 6). It operates in plastids and contributes to the formation of monoterpenes, linalyl acetate, sesquiterpenes, diterpenes, carotenoids, and phytols [36]. Importantly, pyruvate level increased after GXS16 treatment, suggesting a high rate of energy generation due to ATP and NADH production under both aerobic and anaerobic conditions [37].

In sugarcane, GGPP produces prephytoene diphosphate and converts it to phytoene under the regulation of crtB (Fig. 6). Subsequently, the synthesis of astaxanthin and lutein was found to be regulated by crtZ in the astaxanthin and lutein biosynthesis pathways. In addition, beta-carotene was transformed into higher-order building blocks: beta-cryptoxanthin, zeaxanthin, antheraxanthin, violaxanthin, ABA, 8'-hydroxyabscisate, phaseic acid, and dihydrophaseic acid. We inferred that the high levels of ABA in the ID group (Fig. 2E) were strongly linked to the upregulation of CYP707A during ABA biosynthesis (Fig. 6). Carotenoids serve as accessory pigments that harvest light for photosynthesis and constitute the basic structural units of the photosynthesis apparatus [38]. However, they also have essential photoprotective roles; they scavenge ROS, quench the dangerous triplet states of chlorophyll, and participate in the thermal dissipation of excess light energy [39]. Thus, downregulation of astaxanthin, lutein, and dihydrophaseic acid may disrupt photosynthesis when ABA plays an active role in the stress response.

## Conclusion

In conclusion, we found that normal or strong sugarcane growth under drought stress can be assisted by the *Burkholderia* endophytic nitrogen-fixing bacterium GXS16. Multiple DEGs that are potentially associated with sugarcane growth and drought tolerance were identified. In particular, the upregulation of carotenoid and terpenoid backbone biosynthesis in sugarcane may provide new insights into the molecular mechanisms of drought tolerance. Besides, crtB, crtZ, CYP707A, dxr, and PCME got involved in drought resistance function. These findings provided promising candidate genes for the subsequent genetic improvement of sugarcane drought response and may play a critical role in the development of molecular breeding. Compared with wild-type sugarcane, these novel varieties response better to adverse factors and maintain sugar accumulation.

## Methods

### Plant material and sampling

The sugarcane variety ROC16 was provided by the Sugarcane Research Institute of the Guangxi Academy of Agricultural Sciences (Guangxi Zhuang Autonomous Region, China). The endophytic nitrogen-fixing bacterium GXS16 was previously isolated from the sugarcane variety Guitang 31 by our research team [40]. According to the detected amount of protein and α-ketobutyrate, the ACC deaminase activity of GXS16 was determined using the 2,4-dinitrophenylhydrazine method using acdS-specific PCR primer pair: Forward sequence (5'-ATCGGCGGCATCCAGWSNAAYCANAC-3') and Reverse sequence (5'-GTGCATCGACTTGCCCTCRTANACNGGRT-3').

GXS16 cells were inoculated as previously described [41]. Briefly, 100 µl bacterial suspension (1–2 × 10^8^ CFU mL^−1^) was applied to the plantlets in liquid one-tenth MS medium (without vitamins or plant hormones), and another 300 ml bacterial suspension was used to irrigate the plant roots at 15 days after transplanting into pots. Plants in the control group were treated with an equivalent volume of sterilized water at the same time points.

In this study, four experimental treatments were designed: 1) a control (C) group under normal watering conditions, which was maintained under a continuous supply of 200 ml sterile water per day; 2) an inoculation control (IC) group, which was inoculated with GXS16 and grown under normal watering conditions; 3) a drought (D) group, which was not watered throughout the experimental stages from the time point of the inoculation procedure; 4) an inoculation drought (ID) group, which was not watered following inoculation with GXS16. The roots of the plants were collected at 3 days (D1), 5 days (D2), and 7 days (D3) after water deficit (corresponding to mild, moderate, and severe drought stress, respectively), and then frozen in liquid nitrogen for the determination of physiological indices and transcriptome sequencing. Three biological replicates were used for each drought stage and each treatment group.

Genomic DNA was extracted from root samples using the CTAB method, and bacterial DNA was amplified using TaqMan quantitative PCR (Life Technologies, USA) with primers (Forward sequence 5’-GCAGGCGGTTTGCTAAGACC-3, Reverse sequence 5’- GCTTTCGTGCATGAGCCGTCA-3,’ and probe sequence, 5’-CGGGCTCAACCTGGGAACTGC-3’). All reactions were performed using the SYBR Green PCR Master Mix (Takara). The copy numbers of GXS16 were calculated from Ct values based on a plasmid DNA standard curve. Three replicates were performed for each stage and treatment group.

### Physiological measurements

The abundance of Pro, MDA, and H_2_O_2_ in the roots was determined according to the methods of Bates, Heath, and Jaleel et al. [42–44]. ABA and GA3 levels were determined as described previously [45]. CAT, SOD, APX, and GR activities were determined using commercial kits from Suzhou Keming Biotechnology Co., Ltd. following the manufacturer’s instructions. Three replicates were analyzed.

### RNA extraction and RNA-seq analysis

Total RNA was isolated from sugarcane roots using the Qiagen RNeasy Mini Kit (Qiagen) following the manufacturer’s protocol. RNA quantity and quality were assessed using a NanoDrop 2000 (Thermo Scientific, USA) and an Agilent 2100 Bioanalyzer (Agilent Technologies, USA). RNA was treated with DNase I and reverse-transcribed into first-strand cDNA using random hexamer primers. The second-strand cDNA was synthesized using a deoxynucleoside triphosphate (dNTP) mixture containing dUTP. A paired-end library was constructed following end repair, adaptor joining and purification of the DNA fragments. The cDNA library was then sequenced using BGISEQ‐500 (BGI, Shenzhen, China). Three biological replicates were used for each drought stage and each treatment group.

Quality control of raw reads and adapter trimming were performed using fastp (v0.23.2) [46]. The clean reads were aligned to the reference sugarcane genome (NCBI accession: ASM2245720v1) using HISAT2 [47] and quantified using feature counts (SUBREAD software v2.0.1) [48]. Differential expression analysis was performed using DESeq2 (v1.36.0) [49], and the genes with a false discovery rate (FDR) < 0.05 and |log2(fold change)|≥ 1 were defined as significant differentially expressed genes (DEGs). KEGG pathway enrichment analysis of DEGs from all comparisons between groups and stages was performed using the ClusterProfiler package (v. 4.4.4) [24, 50]. Pathways with a *P* value < 0.05 were considered significantly enriched.

### Temporal analysis

Time-series analysis was performed using short time-series expression miner (STEM) v1.3.13 [51]. Genes were clustered across different drought stress stages in each experimental group (C, D, IC, and ID). Significant STEM profiles (*P* < 0.05) were identified and divided into clusters with different trends in each group. Unique and common genes with a particular trend in each group were counted and subjected to KEGG pathway enrichment analysis as described above [24, 52].

### Weighted correlation network analysis

A weighted correlation network analysis (WGCNA) was performed using the WGCNA package (version1.72.1) [53]. To filter out the constantly or lowly expressed genes, those with an averaged reads per kilobase per million (RPKM) ≥ 0.3 were retained for the subsequent identification of WGCNA modules with the parameters of “softPower = 20, cutHeight = 0.7, and minModuleSize = 50”. The module-trait relationship was obtained by relating eigengenes from each module to traits such as experimental time points, bacterial GXS16 treatment conditions, and drought stress conditions using Pearson correlation analysis. Genes in modules with significant module-trait associations (*P* < 0.05) were used for KEGG enrichment analysis as described above [24].

### Statistical analysis

GXS16 copy numbers were statistically compared between different time points and groups using Student’s t-test. Plant height, RWC, levels of H_2_O_2_, Pro, MDA, ABA, SA, GA3, and IAA, and CAT, SOD, APX, and GR activities were statistically compared between different time points and groups using one-way ANOVA and Duncan’s multiple range test.

### Supplementary Information

**Additional file 1: Figure S1.** Copy number of GXS16 in sugarcane root across drought stages. **Figure S2. **Temporal profile analysis of genes across drought stages for C (A), D (B), IC (C), and ID groups (D) by STEM respectively.

**Additional file 2: Table S1.** Plant height (in centimeters) was assessed for distinct sugarcane groups under varying drought stages, with a total of 12 replicates measured. **Table S2.** RNA-Seq read and mapping statistics. BF and AF represent the reads before and after quality filtering, respectively. **Table S3.** Number of DEGs. **Table S4.** Number of distinct trend genes in each WGCNA modules. C_IC_gene indicates the genes which show a particular trend across drought stress stages in IC group compared with C group. D_vs_ID indicates the genes which show a particular trend across drought stress stages in ID group compared with D group. ID_trend_diff indicates the specific trend gene set which show a particular trend across drought stress stages in ID group compared with C, D, and ID groups. **Table S5.** KEGG pathway enrichment analysis of the genes in honeydew module.

## Data Availability

The datasets generated and analysed during the current study are available in the NCBI SRA repository (https://www.ncbi.nlm.nih.gov/bioproject/PRJNA951683).

## Abbreviations

C: Control
IC: Inoculation Control
D: Drought
ID: Inoculation Drought
Pro: Proline
MDA: Malondialdehyde
H_2_O_2_: Hydrogen peroxide
SOD: Superoxide dismutase
APX: Ascorbate peroxidase
GR: Glutathione reductase
ABA: Abscisic acid
WGCNA: Weighted correlation network analysis
RWC: Relative water content
SA: Salicylic acid
IAA: Indole-3-acetic acid
CAT: Activities of catalase
ANOVA: Analysis of variance
DEG: Differentially expressed gene
RPKM: Reads per kilobase per million
DAM: Differentially accumulated metabolites
ACC: 1-Aminocyclopropane-1-carboxylate
