# Supplementary material for: Regulation of an endophytic nitrogen-fixing bacteria GXS16 promoting drought tolerance in sugarcane
Source: BMC Plant Biol. 2023 Nov 17;23:573. doi: 10.1186/s12870-023-04600-5 (PMC10655487; doi:10.1186/s12870-023-04600-5)
Supplement: Supplementary file 1 — Additional file 1: Figure S1. Copy number of GXS16 in sugarcane root across drought stages. Figure S2. Temporal profile analysis of genes across drought stages for C (A), D (B), IC (C), and ID groups (D) by STEM respectively. [file 12870_2023_4600_MOESM1_ESM.docx]

**Supplementary figures for**Regulation of an endophytic nitrogen-fixing bacteria GXS16 promoting drought tolerance in sugarcane

Qian Nong1, 2†, Li Lin1†, Jinlan Xie1, Zhanghong Mo1, Mukesh Kumar Malviya1, 3, Manoj Kumar Solanki4, Zeping Wang1, Xiupeng Song1, Yangrui Li1* and Changning Li1*

*Corresponding author E-mail: liyr@gxaas.net; lcn560@126.com

**This file includes:**

**Supplementary Figures 1 to 2**

**
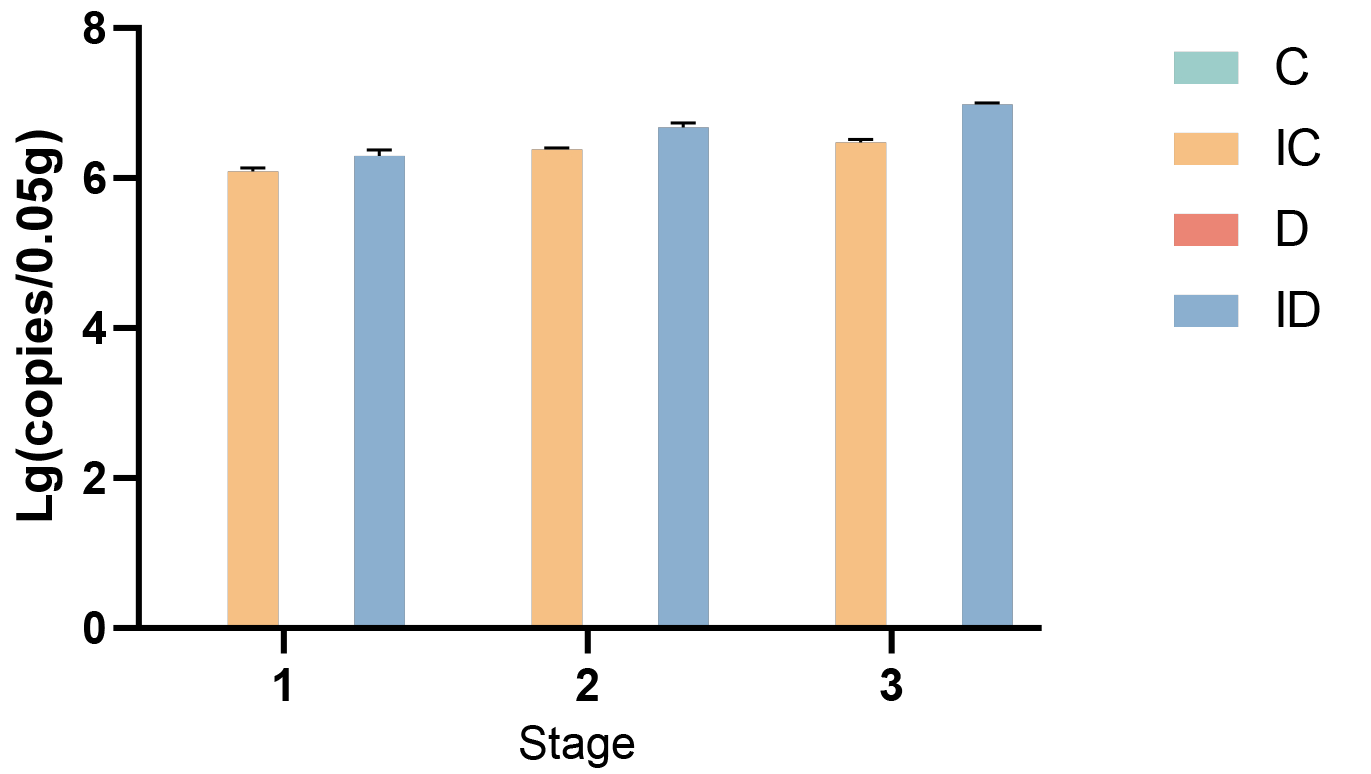
**

**Fig. S1.** Copy number of GXS16 in sugarcane root across drought stages.

**Fig. S2.** Temporal profile analysis of genes across drought stages for C (A), D (B), IC (C), and ID groups (D) by STEM respectively.
